# Healthcare-seeking behaviour of primary caregivers for acute otitis media in children aged 6 months to <30 months in Panama: results of a cross-sectional survey

**DOI:** 10.1186/s12887-016-0760-1

**Published:** 2017-01-05

**Authors:** Iris Villarreal, Rosario Turner, Hyejin Jo, Julie Park, Eric Gemmen, Jean-Yves Pirçon, Maria M. Castrejon, William P. Hausdorff

**Affiliations:** 1 Cromsource for GSK, Avenue Fleming 20, W23 B2-183, 1300 Wavre, Belgium; 2 Caja de Seguro Social de Panamá, La Chorrera, 507 Panama City, Panama; 3 Quintiles Real-world & Late Phase Research, 201 Broadway, Cambridge, MA 02139 USA; 4 Quintiles Real-world & Late Phase Research, 1801 Rockville Pike, Rockville, MD 20852 USA; 5 GSK, Avenue Fleming 20, 1300 Wavre, Belgium; 6 GSK, City of Knowledge, 230 BLDG, Panama City, Panama

**Keywords:** Acute otitis media, Ear infection, Survey, Caregivers’ behaviour, Healthcare utilization, Healthcare facility, Severity of illness, Childhood

## Abstract

**Background:**

Acute otitis media (AOM) is the most common bacterial childhood infection. However, caregivers with children having mild episodes often do not seek healthcare services, which may lead to an under-appreciation of the disease experienced by the community. The objectives of this survey were to estimate the proportion of primary caregivers who went to a healthcare facility when they suspected that their child aged 6 to <30 months was having an AOM episode during the past 6 months and to assess what factors influenced their decision.

**Methods:**

This observational, cross-sectional survey of primary caregivers (≥18 years), with at least one child aged 6 to <30 months was performed in 19 healthcare facilities in Panama (March to May 2013). A 28-item paper questionnaire was administered to assess demographic data, AOM symptoms, as well as potential healthcare-seeking behaviour and factors influencing this behaviour. Potential confounding effects were individually assessed using Chi-squared or Cochran-Mantel-Haenszel tests, and all together in logistic regression models.

**Results:**

The total number of eligible participants was 1330 (mean age 28.5 ± 8.0 years). Of these, 245 participants had at least one child whom they suspected had an AOM episode during the past 6 months. Of the 245 participants, 213 (86.9%) sought healthcare at a facility. Several factors were associated with healthcare usage: perceived severity of illness (*p* = 0.001), occupational status of the caregiver (*p* = 0.002), household income (*p* = 0.016) and length of time since the last suspected AOM episode (*p* = 0.032).

**Conclusions:**

When confronted with a child with obvious symptoms of AOM, the majority of caregivers reported seeking healthcare. This behaviour appeared to be associated with factors related to the severity of the illness, the length of time since the last episode, as well as with the income and occupational status of the caregivers themselves. As many episodes of AOM present with non-specific respiratory symptoms, our results apply only to caregivers who were confronted with children with an obvious symptom.

**Electronic supplementary material:**

The online version of this article (doi:10.1186/s12887-016-0760-1) contains supplementary material, which is available to authorized users.

## Background

Acute otitis media (AOM) is the most common bacterial childhood infection; it is estimated that by the age of 2 years, about 70% of children will have had at least one episode of AOM [1]. Although AOM is often treated with antibiotics, children with mild episodes may not reach a healthcare facility for a variety of reasons, including the caregiver’s choice to self-treat the child with home remedies or use over-the-counter antibiotics in countries where these are available, especially for caregivers with prior experience of an AOM episode [2–5]. Other reasons include the overlap of symptoms with other common respiratory illnesses, which may prevent caregivers from recognizing AOM, as well as the expectation of caregivers not to receive antibiotics in areas that practice watchful waiting [6]. This may lead to an under-estimation of disease incidence in the community if only facility-diagnosed AOM cases are considered.

The efficacy of the ten-valent pneumococcal non-typeable *Haemophilus influenzae* protein D conjugate vaccine against clinically-confirmed AOM in young children was assessed in the Clinical Otitis Media and Pneumonia Study (COMPAS) [7] in a population with good access to healthcare. In the COMPAS study, we captured substantially fewer AOM cases than expected. Various factors are speculated to have contributed to this outcome. Amongst others, enhanced surveillance was introduced late during the trial, after children had already passed the age of peak incidence for AOM. Also, the requirement for evaluation by a paediatrician and further assessment by an ear, nose and throat specialist, as well as the reluctance of seeking specialised care for caregivers perceiving AOM as mild could have prompted them to receive care from pharmacies or non-clinical trial clinics.

As it is unknown to which extent these factors may have played a role, this cross-sectional, survey-based study attempts to assess one of these issues by determining whether under-utilization of healthcare facilities could have contributed to the low incidence of AOM observed in the COMPAS study [7]. The aim is to allow a better understanding of healthcare seeking behaviour amongst caregivers when their children aged 6 months to <30 months experience at least one obvious AOM symptom. To this end, the primary objective was to estimate the proportion of primary caregivers who, over the last 6 months, reported visiting a healthcare facility when they were likely to suspect their child aged 6 to <30 months to have AOM. Secondary objectives included: estimating the proportion of primary caregivers with at least one child they were likely to suspect to have had AOM, determining the healthcare-seeking behaviour of primary caregivers by clinical trial site status and public versus private site status, and identifying the potential determinants influencing the healthcare-seeking behaviour of primary caregivers when they were likely to suspect their child to have AOM.

## Methods

### Study design

This observational, cross-sectional, survey-based study was conducted in 19 healthcare facilities in urban areas of Panama between 27 March and 15 May 2013 and was based on caregiver recall of suspected AOM episodes and healthcare-seeking behaviour that occurred in the previous 6 months. Half of the sites that participated in this survey took part in the COMPAS study, the other half was matched by facility type, location, population coverage and number of subjects that could be recruited in the time of our study. Originally, there were 18 sites planned for study recruitment, but one site dropped prior to study enrolment. Therefore, two additional back-up sites were added which resulted in the total of 19 participating sites.

Clinics were stratified into ‘former clinical trial sites’ and ‘non-clinical trial sites’, based on their past participation in the COMPAS trial. In the case of this study, the ‘former clinical trial sites’ had already participated in the COMPAS trial. A pool of ‘non-clinical trial sites’, located in the same province, was identified by convenience sampling (i.e. geographical accessibility and ease of access to statistical data). These were matched to the ‘former clinical trial sites’ by facility type, location, and size of eligible patient population for this study. With the incorporation of this matching, the primary difference between ‘former clinical trial sites’ and the “non-clinical trial sites” was simply that the former had previously participated in the COMPAS clinical trial and were potentially more research-savvy. The “former clinical trial sites” had originally been chosen for participation in that trial because the local investigators associated with them were previously experienced in epidemiological surveillance research, including participation in an international surveillance study of invasive bacterial isolates in Panama City. Importantly, both ‘former clinical trial sites’ and ‘non-clinical trial sites’ included private and public facilities. For all sites, eligible participants were enrolled on an on-going basis, in proportion to the population coverage of each site.

### Participants and survey

Primary caregivers were approached with a face to face survey by on site physicians during the selected time period if the investigator believed they could comply with the inclusion criteria. The possible participants were mostly caregivers who would come for healthcare for their children and who should have been of legal age (18 years or older), even though it was not a requirement for the specific child in question to be present at the moment of the survey. They received explanation of the consent proceeding and if they agreed to participate, they were asked for a document that would confirm that they were 18 years of age or older. Information on demography, household characteristics, healthcare practices, socio-economic status, and perception of illnesses was solicited. A primary caregiver was defined as an individual who attends to the medical needs and has made healthcare decisions for a child in the last 6 months. This person could be a parent, foster parent, grandparent, another relative, or another responsible adult. Primary caregivers who were likely to suspect their child to have AOM during the past 6 months were further questioned on symptoms observed during the most recent episode and healthcare practices for that episode. The types of healthcare facilities visited by the study participants who were likely to suspect their child had AOM included polyclinics. A polyclinic is a health centre of the social security system that provides preventive, curative and rehabilitative services and employs specialists of various disciplines including generalists, paediatricians, surgeons, interns, orthopaedists, nurses, social workers, laboratory technicians, and radiologists.

The paper-based study questionnaire (Additional file 1) included 28 items and was administered at the enrolment site by trained physicians as a face to face survey. The health care seeking behaviour questions were initially formulated based on an informal literature review. The appropriateness and specific questions were modified based on the specifics of the study, refined and validated in meetings with local paediatricians and investigators, and piloted in a few centres to ensure comprehension before finalisation of the questionnaire. Several questions allowed for multiple responses (for example, the reason for not seeking a healthcare facility when they were likely to suspect that their child had AOM). Questionnaire results were entered weekly by Quintiles into an online survey collection tool. Completed paper questionnaires were sent to a central site where surveys were entered into a database using the KeySurvey software by one person. There was one data entry audit in which 25% of questionnaire data from 3 sites were checked to make sure they were entered accurately, and the audit results showed a less than 5% error rate.

Participants were included in the study if they were 18 years or older, and were the primary caregivers of at least one child aged 6 months to <30 months at the time they were visiting the clinic. Primary caregivers were included in the study regardless of whether their child was suspected to have had AOM or not, but it should be noted that no physician or other healthcare provider-based diagnosis of AOM was necessary for inclusion in the present study. If more than one primary caregiver fulfilling the inclusion criteria was present per child, only one was enrolled in this study. Caregivers who had previously already answered the survey were excluded from further participation. The index child of the caregiver did not need to be present at the time of the survey, and this information was not recorded.

We considered a child suspected of AOM if the primary caregivers reported the presence of at least 2 of the 4 following symptoms (3 of which are directly linked to the ear): (a) ear ache or ear pain; (b) ear discharge; (c) ear rubbing or tugging; (d) general respiratory symptoms (runny nose, sore throat, or cough), and at least one of the following eight additional health events: hearing problems; fever; tiredness or lack of energy; decrease in appetite; vomiting; diarrhoea; trouble sleeping; irritability or excessive crying. These cases are referred to as “suspected AOM” in the result section.

Symptoms considered indicative of AOM were those symptoms used in the COMPAS trial that caregivers could assess by themselves and from which it could reasonably be suspected that the child had AOM (the COMPAS trial also included otoscopic examinations, but they were not included here) [7].

### Sample size

Sample size was determined using a standard formula [8], based on an annual estimated incidence of AOM in children aged 1–4 years of 60% [1] and an assumption that 50% of caregivers would utilize healthcare facilities for suspected AOM episodes. We used the Clopper-Pearson model to obtain a 95% 2-sided confidence interval (CI) with a width of ±5%, and a required sample size of 1300 participants was calculated.

### Statistical analysis

All study questionnaires were included in the statistical analysis regardless of whether they were fully completed or not, unless consent was withdrawn by the participant. All performed analyses were based on the total number of eligible participants.

The potential confounding effects of demographic characteristics and other covariates of interest on the study outcomes were assessed individually using the Chi-squared or the Cochran-Mantel-Haenszel test, and all together in logistic regression models.

All statistical analyses were carried out using SAS 9.2. *P*-values ≤0.05 were used as an indicator that a difference between groups may exist.

## Results

### Study population

Demographic characteristics of caregivers included in the study and of caregivers whose child had a suspected AOM episode are summarized in Table 1, and the number of responders for different questions is given as Additional file 2: Figure S1.

**Table 1:** Characteristics of primary caregivers enrolled in the study

Characteristics of primary caregiver	Categories	Value or proportion (%)^a^
Total enrolled participants		*N* = 1330
Age (years)	Mean (SD)	28.5 (7.98)
	Range	18–67
Gender	Male	69/1324 (5.2)
	Female	1255/1324 (94.8)
	Missing	6
Highest education level	Less than University^b^	873/1330 (65.6)
	University	457/1330 (34.4)
Occupation	Housewife	725/1330 (54.5)
	Student	30/1330 (2.3)
	Employed part-time	71/1330 (5.3)
	Employed full-time	477/1330 (35.9)
	Unemployed	13/1330 (1.0)
	Retired	1/1330 (0.1)
	Other	13/1330 (1.0)
Number of children	1	586/1329 (44.1)
	2	443/1329 (33.3)
	3	191/1329 (14.4)
	>3	109/1329 (8.2)
	Missing	1
Public/private status	Public	1150/1330 (86.5)
	Private	180/1330 (13.5)
Trial/non-trial status	Trial	826/1330 (62.1)
	Non-trial	504/1330 (37.9)
Primary caregivers whose child had a suspected AOM episode		*N* = 245
Household members^c^	Other children <5 years old [median (min-max)]	1 (0–8)^d^
	Children 5–18 [median (min-max)]	1 (0–7)
	Adults >18 years old [median (min-max)]	2 (0–10)
Overall income/month (US$)^c^	<400	64/244 (26.2)
	400–999	86/244 (35.2)
	1000–1999	48/244 (19.7)
	>2000	12/244 (4.9)
	Unknown/prefer not to answer	34/244 (13.9)
	Missing	1
Medical insurance^c^	Public	119/238 (50.0)
	Private	2/238 (0.8)
	Public and private	10/238 (4.2)
	No	107/238 (45.0)
	Missing	7
Day-care attendance^c^	Yes	22/244 (9.1)
	No	222/244 (91.0)
	Missing	1
Relationship to child^c^	Parent	228/243 (93.8)
	Grandparent	8/243 (3.3)
	Other relative	7/243 (2.9)
	Missing	2
Regular visit to doctor^c^	Yes	127/244 (52.0)
	No	117/244 (48.0)
	Missing	1

*AOM* acute otitis media, *N* total number of participants in ATP cohort or participants whose child had a suspected AOM episode, *Value* value of the considered parameter, *Proportion (%)* proportion (percentage) of participants in a given category, *SD* standard deviation

^a^Proportions for each response category do not include missing responses

^b^Less than University includes No School, Primary School, Secondary School and Vocational School

^c^These variables are defined only for the participants whose child had a suspected AOM episode

^d^The question used to collect information about household members < 5 years may have been misinterpreted and some of the caregivers may have included the child for which symptoms where described into their counting

Of the 1332 primary caregivers enrolled in the study, 2 reported that they did not have a child aged 6 to <30 months and were withdrawn from the study. As a result, the total number of eligible participants was 1330, with a mean age (± standard deviation) of 28.5 (±7.98) years. Information regarding the number of caregivers who were approached but did not consent to the study was not collected systematically, but a post-hoc telephone survey of coordinators of 8 sites, representing around 700/1330 forms included in the study, suggested that more than 90% consented to participate. The majority of primary caregivers for whom data regarding gender were available were female (1255/1324, 94.8%). Over a third of caregivers had a University degree (457/1330, 34.4%) and most had either one (586/1329, 44.1%) or two children (443/1329, 33.3%).

When asked about the reason for using a healthcare facility at the moment of the questionnaire, more than half of caregivers answered wellness visit (764/1332, 57.8%), while over a third were attending due to a sick visit (38.0%). Of the 1330 eligible caregivers, 1329 caregivers answered at least one of the two questions on health problems used to define the cases suspected of having an AOM episode sometime in the previous 6 months. The majority of these caregivers reported that their child aged 6 to <30 months had runny nose, sore throat or cough (82.4%). The most commonly reported additional health problem was fever (55.2%). Of these 1329 caregivers, 245 (18.4%) had at least one child that had a combination of symptoms during the previous 6 months that we consider likely indicative of an AOM episode, answering positively for at least 3 concomitant symptoms or health problems as described in the Methods.

### Healthcare-seeking behaviour of caregivers having at least one child with a suspected AOM episode

The majority of caregivers who had at least one child with a suspected AOM episode reported that they sought healthcare at a facility (213/245, 86.9%). Most caregivers went to one of the following: a public health centre (39.9%), a private doctor’s facility (32.9%), a public hospital (27.7%), or a polyclinic (17.8%).

Of the 213 caregivers who had visited a healthcare facility when their child had a suspected AOM episode, 84.5% went to a public rather than a private healthcare facility; 63.8% went to one of the former COMPAS trial sites. Caregivers showed no preference for using a private versus public or former versus non-COMPAS site.

**Fig. 1:**
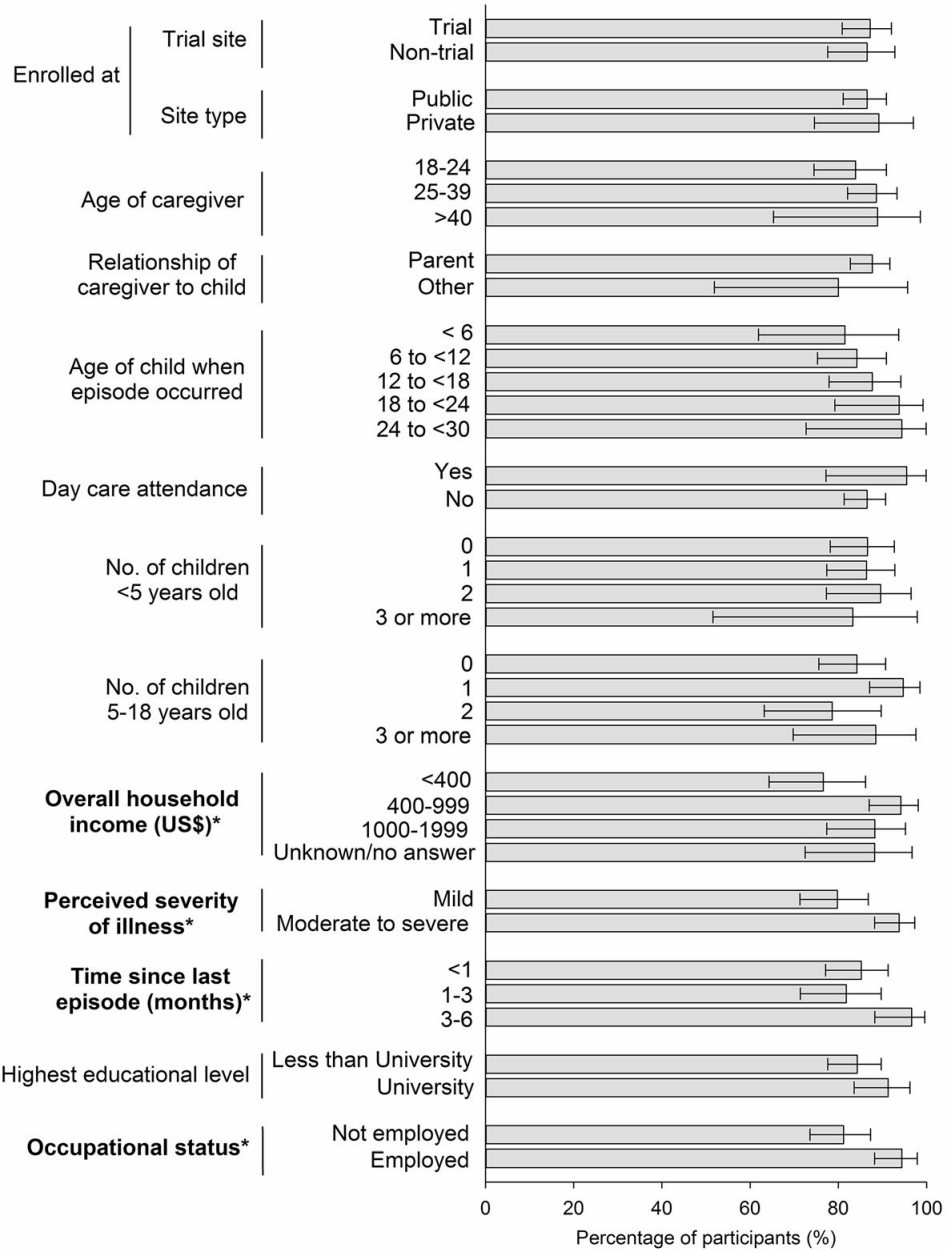
Comparison between the percentages of participants who used a healthcare facility when they suspected AOM. The percentages presented are based on the number of primary caregivers who used a healthcare facility divided by the total number of primary caregivers whose child were suspected of AOM within a specific category of factor. AOM = acute otitis media. “Trial Site” refers to sites that have previously participated in the COMPAS trial. Error bars indicate 95% confidence intervals. *Factors which are found to be statistically different for the use of healthcare facilities, using the Chi-Squared test at alpha = 0.05 for variables with 2 categories and Cochran-Mantel-Haenszel test for variables with more than 2 categories

**Fig. 2:**
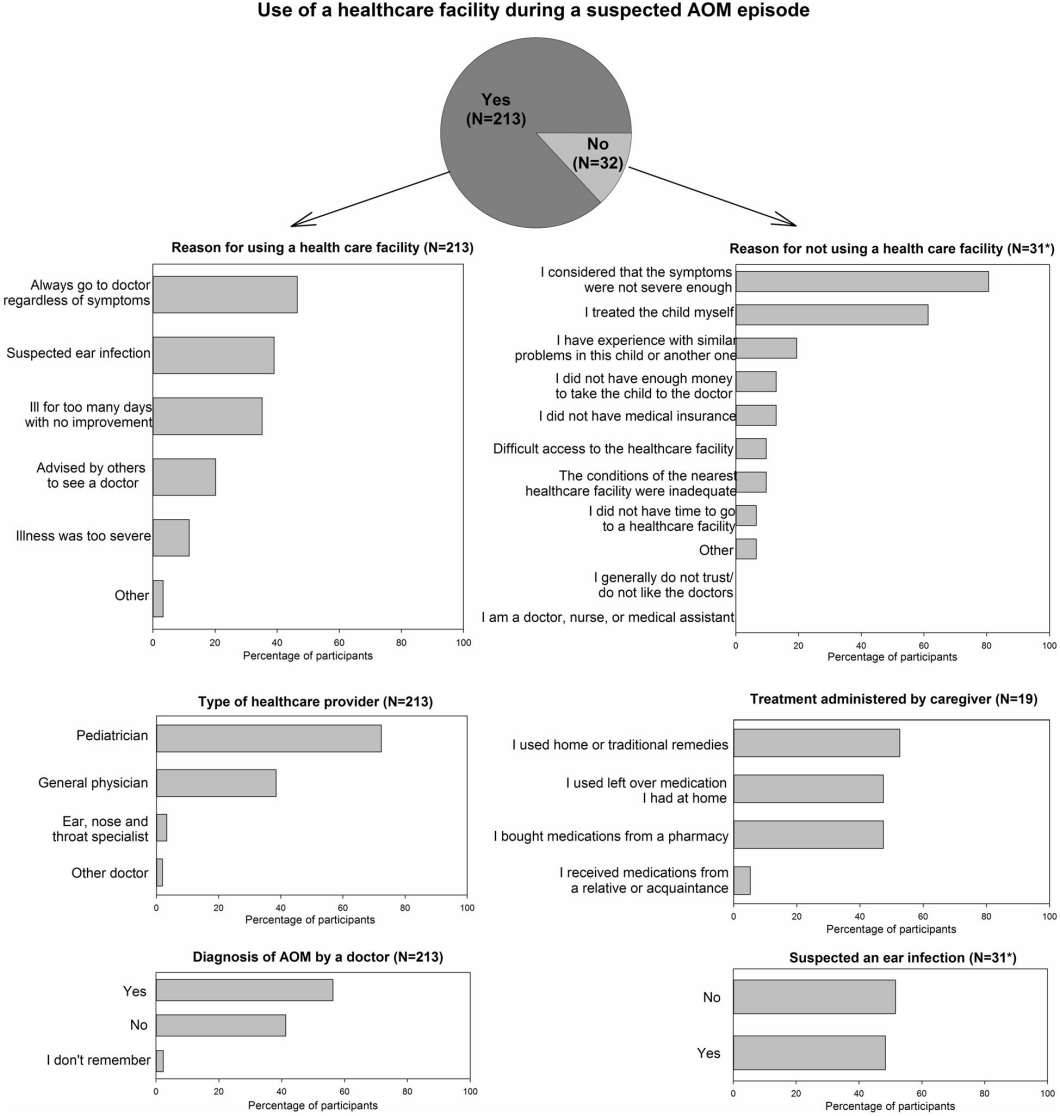
Healthcare seeking behaviour characteristics in caregivers whose child was suspected of having an AOM episode. AOM = acute otitis media. *N* = total number of participants in a given category. *Number of participants who answered the question

Caregivers with suspected AOM cases were asked to provide one or more reasons for going or not going to a healthcare facility. Among the 213 caregivers who sought healthcare at a facility, the most frequently cited reason was “I always go to doctor regardless of the symptoms” (46.5%), followed by “suspected ear infection” (39.0%). The majority of those who used a healthcare facility went to a paediatrician (72.3%). According to the primary caregivers’ answers, over half (56.3%) of the children with a suspected AOM episode were diagnosed with an ear infection by the healthcare provider (Fig. 1). Among the 32 caregivers who did not seek healthcare at a facility, almost all (31, 97%) provided motivation for their choice, but of these half (16/31, 52%) did not suspect that their child had an ear infection, despite the presence of at least one ear symptom (Fig. 2). The most frequently cited reason for not using a healthcare facility was “I considered that the symptoms were not severe enough” (81%), followed by “I treated the child myself” (19/31, 61%). These 2 reasons were given either individually or together by the majority of caregivers (97%). Over half of the caregivers who treated their children themselves reported “I used home or traditional remedies” (10/19, 53%). The other 2 main reasons were “I bought medications from a pharmacy” (47%) and “I used leftover medication I had at home” (47%).

### Factors influencing healthcare-seeking behaviour

Several factors were evaluated for their potential effects on the caregivers’ healthcare-seeking behaviour. Based on the comparison of proportions, 4 factors were found to have a statistically significant effect on healthcare-seeking behaviour: (i) perceived severity of illness (*p* = 0.001), (ii) occupational status of the caregiver (*p* = 0.002), (iii) household income (*p* = 0.016) and (iv) length of time since the suspected AOM episode occurred (*p* = 0.032) (Fig. 1).

Logistic regression models showed similar results (Table 2): caregivers with a monthly household income between $400 and $999 had higher odds of using a healthcare facility compared to those who earned < $400 (adjusted odds ratio [OR] 5.6, 95% CI 1.4–22.0); other factors that increased the odds of going to a healthcare facility were the caregiver’s perception of the illness as moderate or severe, compared to mild (adjusted OR 5.5, 95% CI 2.0–15.5), and the employment status of the caregiver (adjusted OR 3.3, 95% CI 0.9–11.7). Caregivers whose child had an episode that had occurred 3–6 months before the survey had higher odds of having gone to a healthcare facility compared to those whose child’s episode occurred less than a month before the survey (adjusted OR 6.66, 95% CI 1.3–34.4). The same 4 factors were found to have a potential effect on healthcare facility usage when a logistical regression model with backward selection was employed (significance level of 0.05, Table 2).

**Table 2:** Adjusted odds ratio (OR) of AOM episodes suspected by primary caregivers

Logistic Regression Model		
Factor		Adjusted OR (95% CI)
By trial site	Non-trial sites	Reference
	Trial site^a^	1.08 (0.39, 3.02)
By type of sites	Public	Reference
	Private	1.08 (0.25, 4.74)
By age (Years) of caregiver	18-24	Reference
	25-39	0.77 (0.25, 2.38)
	≥40	2.05 (0.22, 18.76)
By relationship of caregiver to child	Mother or father	Reference
	Other relatives^b^	0.20 (0.03, 1.41)
By age of child when episode occurred	Less than 6 months	Reference
	6 to less than 12 months	1.14 (0.26, 5.01)
	12 to less than 18 months	2.19 (0.42, 11.30)
	18 to less than 24 months	4.48 (0.46, 43.68)
	24 to less than 30 months	3.23 (0.25, 41.24)
By day care attendance	Yes	Reference
	No	0.92 (0.09, 9.58)
By number of other children in household: <5 years old^c^	0	Reference
	1	0.76 (0.25, 2.33)
	2	1.80 (0.46, 7.06)
	3 or more	2.67 (0.20, 36.51)
By number of children in household: 5–18 years old	0	Reference
	1	2.41 (0.60, 9.61)
	2	0.67 (0.19, 2.39)
	3 or more	1.49 (0.27, 8.10)
By overall income (US dollars)	Less than 400	Reference
	400 to 999	5.63 (1.44, 22.01)
	1000 to 1999	1.20 (0.28, 5.17)
	Unknown or prefer not to answer	2.78 (0.58, 13.33)
By perceived severity of illness	Mild illness	Reference
	Moderate or severe illness	5.53 (1.98, 15.49)
By length of time since episode (months)	Less than one month ago	Reference
	Between one and three months ago	0.76 (0.26, 2.19)
	Between three and six months ago	6.56 (1.25, 34.40)
By highest educational level	Less than University	Reference
	University	0.96 (0.27, 3.46)
By occupational status	Not-employed^d^	Reference
	Employed	3.26 (0.91, 11.67)
Logistic Regression Model with Backward Selection (at 0.05 significance level)		
Factor		Adjusted OR (95% CI)
By overall income (US dollars)	Less than 400	Reference
	400 to 999	4.71 (1.36, 16.37)
	1000 to 1999	1.24 (0.38, 4.02)
	Unknown or prefer not to answer	3.46 (0.966, 12.56)
By occupational status	Not-employed	Reference
	Employed	3.27 (1.08, 9.91)
By perceived severity of illness	Mildly ill	Reference
	Moderately or severely ill	4.61 (1.83, 11.61)
Length of time since episode (months)	Less than one month ago	Reference
	Between one and three months ago	0.70 (0.28, 1.72)
	Between three and six months ago	5.93 (1.24, 28.37)

OR were adjusted for all covariates in a model

No adjustments of CIs were made for the multiplicity of endpoints

*AOM* acute otitis media, *CI* confidence interval

^a^ “Trial Site” refers to sites that have previously participated in the COMPAS trial

^b^ “Other relatives” include ‘step-mother or step-father’, ‘grandmother or grandfather’, ‘other relative’, and ‘none of the above’

^c^ The question used to collect information about household members < 5 years may have been misinterpreted and some of the caregivers may have included the child for which symptoms where described into their counting

^d^ “Not-employed” includes ‘housewife’, ‘unemployed’, ‘retired’, ‘specified as other’, and ‘student’

## Discussion

Among all eligible caregivers who completed the survey, 18.4% had at least one child aged 6 to <30 months that they suspected of having an AOM episode in the past 6 months, and the majority (86.9%) of them went to a healthcare facility. This proportion of almost 87% was higher than expected, based on a previous study in Guatemala [9], which indicated that only approximately one third of children <5 years old with various health problems (respiratory, high fever, diarrhoea, vomiting) were taken to a healthcare facility.

Regarding the percentage of children with suspected AOM, although it is likely that the number of caregivers with at least one child suspected of an AOM episode could have varied over a 12-month period, the 6-month reporting period was chosen to minimise recall bias of the respondents. This bias might have been reduced if we had used a shorter recall period, but having the period extend to 6 months allowed us to maximize chances of capturing more AOM episodes given seasonal variations in incidence.

Implementation of pneumococcal conjugate vaccination prior to the time of the survey could have also affected the frequency with which caregivers observed symptoms typical of AOM. Specifically, a pneumococcal conjugate vaccine (13vCRM; *Prevenar13/Prevnar13*, Wyeth) was added to the universal mass vaccination program in Panama beginning in 2011 [10], administered in a 4-dose schedule during the first 2 years of life, with a single catch-up dose administered to all children between 2 and 9 years of age. Although the impact of vaccination on the percentage of population affected by AOM was not assessed in the present study, introduction of pneumococcal conjugate vaccines in the United States and Europe have led to a decrease in the rate of clinical visits due to AOM [11, 12]. Therefore, we cannot rule out the possibility that the lower-than-expected rate of AOM is partly due to vaccine implementation in Panama.

Another limitation that needs to be taken into consideration is that the present study only describes care-seeking behaviour for children with obvious ear symptoms that can be recognized by the primary caregivers, whereas, according to other studies, the majority of AOM cases might not show these symptoms to the caregiver, such as in the case of toddlers who show only respiratory symptoms. The collection of symptoms, although applicable in all ages, is used to identify AOM cases for which the caregiver might reasonably have suspected AOM. These symptoms may be even more pronounced in older children, and even more likely to be considered AOM. For example, a Finnish study reported that as many as 41% of the children with AOM did not have ear-related symptoms [13]. Additionally, several studies reported that ear pain, the most specific symptom, is only present in 50–60% of children suffering from AOM [13, 14], ear tugging or rubbing is apparent in approximately 40% of children [14], while more general respiratory symptoms can be noticed in 10–85% of cases [14]. Thus, it is likely that only a subset of all AOM cases were included, namely those in children who exhibited obvious ear-related symptoms that could have been easily recognized by the caregivers.

Although only 12.9% of caregivers who did not go to a healthcare facility indicated financial burden as a reason, our results show that those who earned ≥ $400 for overall household income per month were significantly more likely to use healthcare facilities compared to those who earned < $400 per month. In fact, in many developing countries, children from households with better socioeconomic status and/or who are more severely affected by disease are more likely to seek for medical care [15], which may result in underestimation of burden of disease from facility-based surveillance. In Panama, healthcare costs are state-covered for children under 5 years of age and so financial burden should not be an issue, but this burden could manifest indirectly through a day lost at work, which would be more impactful to caregivers in the lower income group. Also, over half of the caregivers who chose not to take their children to a healthcare facility decided to treat their child themselves by using home remedies, leftover medicines or medicines bought from a pharmacy, all of which could be a way of addressing the child’s symptoms without having to lose a day of work.

Healthcare-seeking behaviour also depends on the educational status and cultural beliefs of the population [16, 17]. Trust in physicians as well as the adequacy of the conditions of the healthcare facilities can influence the caregiver’s decision of seeking healthcare when their child is sick. In our study, none of these factors seemed to discourage caregivers from seeking healthcare when their child was suspected of AOM, which can explain the high number of caregivers who sought healthcare for their children.

Caregivers who suspected their child to have experienced an AOM episode more than 3 months before the survey were more likely to have used a healthcare facility than those in the 0–3 months prior to the survey. This finding was unexpected, considering that more recent visits are more likely to be remembered. Seasonality may offer an explanation, as the rainy season, which takes place from May to December in Panama, occurred prior to the 3–6 months period before the study enrolment, while the dry season overlapped with the study period. Children who had experienced the rainy season might have been more likely to have AOM, or they might have had more severe episodes, hence leading to a higher chance of using healthcare facilities [18]. Recall bias could offer another explanation since caregivers might only remember mild episodes of AOM that were very recent, but not those that had occurred more than a few months ago and which would have not led to a healthcare facility visit. This limitation was mitigated by collecting data on when exactly the suspected AOM episode had occurred to have a better understanding of the memory recall period. However, we cannot exclude that caregivers might have been biased towards remembering the more severe episodes for which they went to healthcare facilities, or that they might erroneously attribute episodes that happened more than 6 months prior to the visit to the 3–6 months period.

Another important drawback to highlight is the probability of bias when using the convenience sampling method. In this study, convenience sampling was used to match ‘non-former clinical trial sites’ to ‘former clinical trial sites’ on the basis of facility type, location, and size of eligible patient population to enrol in this study and no other methods such as random or stratified sampling were performed. To mitigate possible bias, an analysis was run to find potential differences. However, no statistical differences were found between ‘former clinical trial sites’ vs ‘non clinical trial sites’ in terms of seeking healthcare facility for a suspected AOM episode.

Although surveys on healthcare-seeking behaviour are ideally conducted in the community [19], this study was conducted in well-baby clinics. Thus, a limitation that needs to be acknowledged is that study participants were only recruited in a healthcare setting. On the other hand, because 85–90% of caregivers in Panama visit these clinics for their child’s immunisations [20–22], enrolled participants were assumed to be representative of the general population. One of the most frequently cited reasons for using a healthcare facility was “I always go to the doctor regardless of the symptoms”. This finding may be corroborated by the fact that sometimes caregivers also bring their child to the well-baby clinic for a preventive visit or a sick visit. In addition, children with special or chronic conditions may have been referred to some of the larger facilities included in the study, which could have contributed to the inclusion of some caregivers who visited the doctor more regularly. Approximately half of the caregivers who had a child whom they suspected of having AOM in the previous 6 months reported that this child regularly visits the doctor for a particular health problem of any type (results not shown). Thus a slight bias due to inclusion of caregivers who were more likely than the general population to seek healthcare when their child was sick cannot be excluded. Contrary to the caregivers included in this study, the participants in the COMPAS trial [7] brought their children solely for the purpose of immunisation.

On the other hand, the fact that the survey was conducted by physicians could have also introduced a bias in the answers of caregivers, i.e., when asked whether they utilised healthcare facilities, caregivers might have been more likely to give an affirmative answer even if in reality they might have not. In the presence of physicians, caregivers might have been more compelled to say that they sought healthcare for their child’s illness than if they were asked by those not affiliated with the healthcare setting [23].

A final limitation is that the p-values and CIs were not adjusted for multiplicity of endpoints, and consequently statistically significant findings must be interpreted with caution.

## Conclusions

A high percentage of primary caregivers in Panama reported that they visited a healthcare facility during the past 6 months when their child aged 6 to <30 months had several symptoms linked with AOM. These results suggest that, for cases that present with potentially recognizable or obvious AOM symptoms, low healthcare facility use is not a probable cause for the low incidence of facility-diagnosed AOM cases encountered in the COMPAS trial. However, the results presented here do not provide information about the likelihood for caregivers to seek healthcare for children with non-specific symptoms that could have ended up being diagnosed with AOM. Also, the limitations discussed above need to be taken into consideration before a definitive conclusion can be drawn.

Several potential factors were shown to have a statistically significant effect on healthcare utilization: perceived severity of the illness, occupational status of the caregiver, household income and length of time since the episode occurred.

## Additional files

Additional file 1: Survey Questionnaire. Title of data: Additional file 1. Survey questionnaire. Description of data: List of questions and guidance used in the survey to assess demographic data, AOM symptoms, as well as potential healthcare-seeking behaviour and factors influencing this behaviour. (PDF 406 kb)
Additional file 2: Figure S1. Number of responses received to the different sections of the survey. Q = question. AOM = acute otitis media. (PPTX 81 kb)

## Abbreviations

AOM: Acute otitis media
CI: Confidence interval
OR: Odd ratio
COMPAS: The Clinical Otitis Media and Pneumonia Study
