# Supplementary material for: Healthcare-seeking behaviour of primary caregivers for acute otitis media in children aged 6 months to <30 months in Panama: results of a cross-sectional survey
Source: BMC Pediatr. 2017 Jan 5;17:3. doi: 10.1186/s12887-016-0760-1 (PMC5217229; doi:10.1186/s12887-016-0760-1)
Supplement: Additional file 1: — Survey Questionnaire. Title of data: Additional file 1. Survey questionnaire. Description of data: List of questions and guidance used in the survey to assess demographic data, AOM symptoms, as well as potential healthcare-seeking behaviour and factors influencing this behaviour. (PDF 406 kb) [file 12887_2016_760_MOESM1_ESM.pdf]

Survey Questionnaire

If the subject has already participated in this survey, please do not continue the interview.

1. Has the subject signed the informed consent form for this study before answering this questionnaire?
    a. Yes
    b. No

    If the subject answered 'No' at Q1, please do not continue the interview.

2. What is the primary caregiver's age?
    a. __ years old

    If the primary caregiver is younger than 18 years old, please do not continue the interview.

    A **primary caregiver** is an individual who attends to the medical needs and has made healthcare decisions for a child in the last six months. This person could be a parent, foster parent, grandparent, other relative, or other responsible adult.

3. Is the subject a primary caregiver of at least one child aged at least 6 months and less than 30 months old?
    a. Yes
    b. No

If the subject answered 'No' at Q3, please do not continue the interview.

4. What is your gender?
    a. Female
    b. Male

5. What is your highest educational level? (Choose only one)
    a. No school
    b. Primary school
    c. Secondary school
    d. Vocational school
    e. University

6. What is your main occupational status? (Choose only one)
    a. Housewife
    b. Student
    c. Employed part-time
    d. Employed full-time
    e. Unemployed
    f. Retired
    g. Other (specify) ____

7. For how many children are you the primary caregiver?
    a. 1
    b. 2
    c. 3
    d. More than 3

8. What is the reason for your visit to this healthcare facility today?
    a. Wellness visit (immunization or scheduled checkup or screening procedures for prevention)
    b. Sick visit
    c. Other (specify) ____

A **primary caregiver** is an individual who attends to the medical needs and has made healthcare decisions for a child in the last six months. This person could be a parent, foster parent, grandparent, other relative, or other responsible adult.

9. In the past 6 months until today, has a child at least 6 months and less than 30 months old for whom you are a primary caregiver had a time when they were ill with any of the following health problems?
    a. Ear ache or ear pain (Y/N)
    b. Ear discharge (Y/N)
    c. Ear rubbing or tugging (Y/N)
    d. Runny nose, sore throat, or cough (Y/N)

If the subject has answered YES to at least two of the options in Q9 PLUS at least one option in Q10, please continue the survey. Otherwise, the survey has finished.
If there was more than one time within the last 6 months when the child had any of the above health problems, think of the most recent episode.

If you are the primary caregiver for more than one child at least 6 months and less than 30 months old who had any of the above health problems, think of the most recent episode in any of these children.

The following questions are about the most recent episode when this child was ill with any of the above health problems within the last six months.

10. During this time, did this child have any of the following additional health problems?
    a. Hearing problems (Y/N)
    b. Fever (Y/N)
    c. Tiredness or lack of energy (Y/N)
    d. Decrease in appetite (Y/N)
    e. Vomiting (Y/N)
    f. Diarrhoea (Y/N)
    g. Trouble sleeping (Y/N)
    h. Irritability or excessive crying (Y/N)

If the subject has answered YES to at least two of the options in Q9 PLUS at least one option in Q10, please continue the survey. Otherwise, the survey has finished.

The following questions are about the most recent episode when this child had any of the previously described health problems within the last six months.

11. How long ago did the episode occur?
    a. Less than one month ago
    b. Between one and three months ago
    c. Between three and six months ago

12. What was the age of the child when this episode occurred?
    a. Less than 6 months
    b. 6 to less than 12 months
    c. 12 to less than 18 months
    d. 18 to less than 24 months
    e. 24 to less than 30 months

13. How many days was the child ill during this episode?
    a. 1 to less than 3 days
    b. 3 to less than 7 days
    c. 7 to less than 14 days
    d. 14 days or more

14. How sick was the child during this episode?
    a. Mildly ill
    b. Moderately ill
    c. Severely ill

15. What did you do to care for this child during this episode? (Choose all that apply)
    a. Nothing
    b. Gave home remedies
    c. Saw traditional or natural healer
    d. Bought medicines from a pharmacy
    e. Went to private doctor's facility
    f. Went to public health center
    g. Went to policlinic
    h. Went to public hospital
    i. Went to private hospital
    j. Other (specify) ______________________________

If the child did not go to a healthcare facility (answers e-i above), skip to Q19.

16. Why did you take this child to a healthcare facility? (Choose all that apply)
    a. Suspected ear infection
    b. Illness was too severe
    c. Ill for too many days with no improvement
    d. Advised by others to see a doctor
    e. Always go to doctor regardless of the symptoms
    f. Other (specify) ______________________________________

17. Who examined this child? (Choose all that apply)
    a. General physician
    b. Paediatrician
    c. Ear, nose, and throat specialist
    d. Other doctor
    e. Not examined

If this child was examined by a doctor,

18. Did the doctor say that this child had an ear infection?
    a. Yes
    b. No
    c. I don't remember

Skip to Q21.

If this child did not go to a healthcare facility during the episode when the previously described health problems were present,

19. Did you suspect that this child could have an ear infection?
    a. Yes
    b. No

20. What were the factors that influenced your decision not to go to a healthcare facility? (Choose all that apply)
    a. I considered that the symptoms were not severe enough
    b. I am a doctor, nurse, or medical assistant
    c. I did not have time to go to a healthcare facility
        i. I had to work
        ii. I was sick too
    d. I generally do not trust, do not like… the doctors
    e. I treated the child myself
        i. I bought medications from a pharmacy
        ii. I received medications from a relative or acquaintance
        iii. I used left over medication I had at home
        iv. I used home or traditional remedies
    f. I have experience with similar problems in this child or another one
    g. Difficult access to the healthcare facility
        i. Travelling time: |__| hours |__|__| minutes
        ii. Transport difficulties (frequency, crowded, comfort…)
    h. I did not have enough money to take the child to the doctor
    i. I did not have medical insurance
    j. The conditions of the nearest healthcare facility were inadequate
        i. Insufficient medical equipment, medicines...
        ii. Insufficient doctor availability
        iii. Healthcare facility not always open
        iv. Too long to wait to be examined
    k. Other, please specify:_______________________________________

The following questions are about the most recent episode when this child had any of the previously described health problems within the last six months.

21. How many other similar episodes were experienced by this child within the last 6 months?
    a. None
    b. 1
    c. 2
    d. 3 or more
    e. I don't remember

22. Before the episode previously described, have you cared for this child or other children with similar health problems?
    a. Yes
    b. No

23. Does this child regularly visit the doctor for a particular health problem of any type?
    a. Yes
    b. No

24. Who lives in this child's household? How many:
    a. Children under 5 years old __
    b. Children between 5 and 18 years old __
    c. Adults over 18 years old __

25. What is your relationship to this child?
    a. Mother or father
    b. Step-mother or step-father
    c. Grandmother or grandfather
    d. Other relative
    e. None of the above

26. Does this child spend time in a daycare center?
    a. Yes
    b. No

27. What is the overall income in this child's household in US dollars per month?
    a. Less than 400
    b. 400 to 999
    c. 1000 to 1999
    d. 2000 or more
    e. Unknown or prefer not to answer

28. Is this child covered by medical insurance? (Choose all that apply)
    a. Yes, public
    b. Yes, private
    c. No
    d. Unknown
