# Supplementary material for: Healthcare-seeking behaviour of primary caregivers for acute otitis media in children aged 6 months to <30 months in Panama: results of a cross-sectional survey
Source: BMC Pediatr. 2017 Jan 5;17:3. doi: 10.1186/s12887-016-0760-1 (PMC5217229; doi:10.1186/s12887-016-0760-1)
Supplement: Additional file 2: Figure S1. — Number of responses received to the different sections of the survey. Q = question. AOM = acute otitis media. (PPTX 81 kb) [file 12887_2016_760_MOESM2_ESM.pptx]

## Slide 1
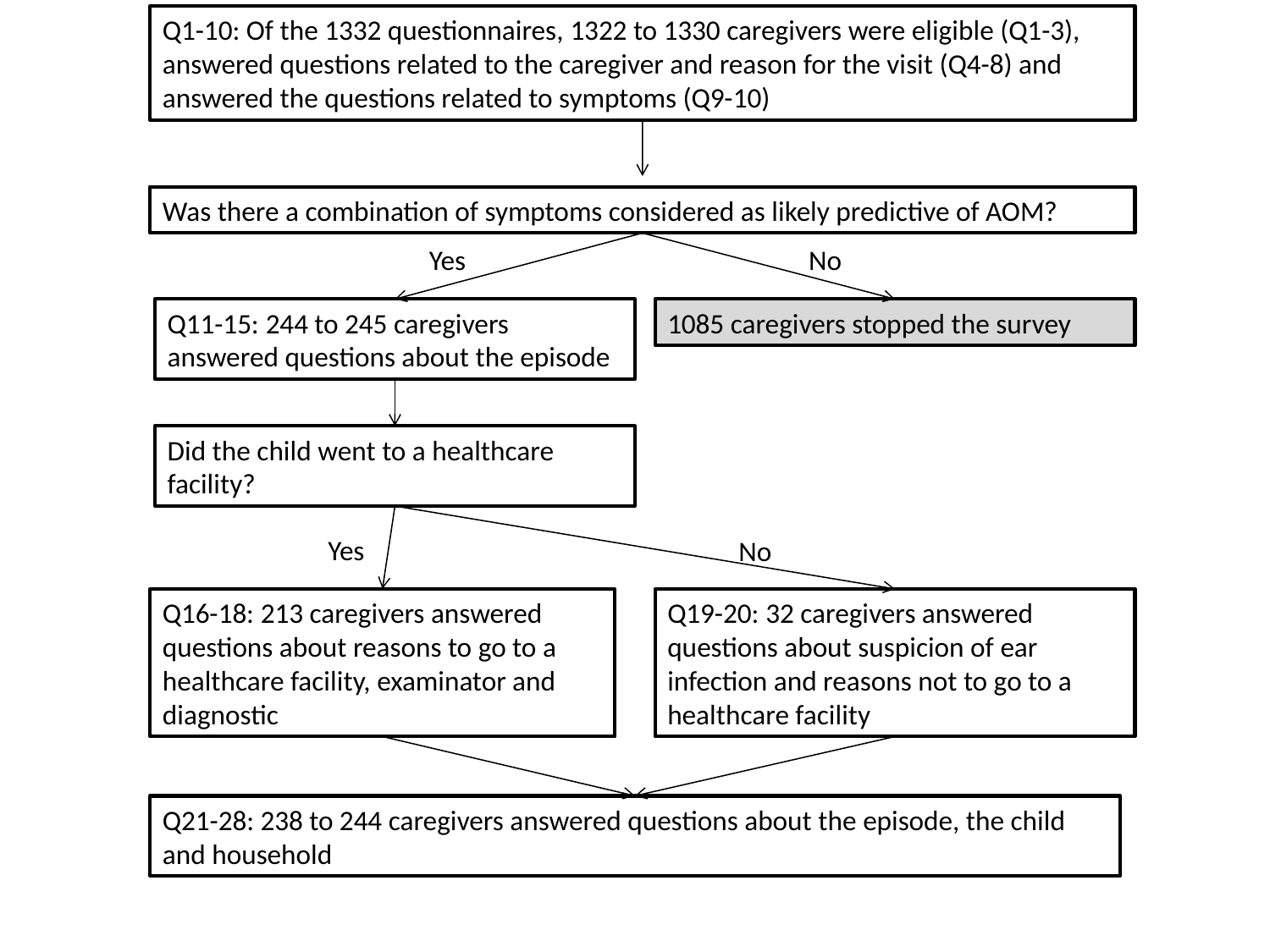

Q1-10: Of the 1332 questionnaires, 1322 to 1330 caregivers were eligible (Q1-3), answered questions related to the caregiver and reason for the visit (Q4-8) and answered the questions related to symptoms (Q9-10)
Was there a combination of symptoms considered as likely predictive of AOM?
Yes
No
Q11-15: 244 to 245 caregivers answered questions about the episode
1085 caregivers stopped the survey
Did the child went to a healthcare facility?
Yes
No
Q16-18: 213 caregivers answered questions about reasons to go to a healthcare facility, examinator and diagnostic
Q19-20: 32 caregivers answered questions about suspicion of ear infection and reasons not to go to a healthcare facility
Q21-28: 238 to 244 caregivers answered questions about the episode, the child and household
